# Hybrid approach of ventricular assist device and autologous bone marrow stem cells implantation in end-stage ischemic heart failure enhances myocardial reperfusion

**DOI:** 10.1186/1479-5876-9-12

**Published:** 2011-01-19

**Authors:** Kyriakos Anastasiadis, Polychronis Antonitsis, Helena Argiriadou, Georgios Koliakos, Argyrios Doumas, Andre Khayat, Christos Papakonstantinou, Stephen Westaby

**Affiliations:** 1 Department of Cardiothoracic Surgery, AHEPA Hospital, Thessaloniki, Greece; 2 Laboratory of Medical Biochemistry, Aristotle University, Thessaloniki, Greece; 3 Nuclear Medicine Department, Aristotle University, Thessaloniki, Greece; 4 Department of Cardiothoracic Surgery, Caen Hospital, Cedex, France; 5 Department of Cardiothoracic Surgery, John Radcliffe Hospital, Oxford, UK

## Abstract

We challenge the hypothesis of enhanced myocardial reperfusion after implanting a left ventricular assist device together with bone marrow mononuclear stem cells in patients with end-stage ischemic cardiomyopathy. Irreversible myocardial loss observed in ischemic cardiomyopathy leads to progressive cardiac remodelling and dysfunction through a complex neurohormonal cascade. New generation assist devices promote myocardial recovery only in patients with dilated or peripartum cardiomyopathy. In the setting of diffuse myocardial ischemia not amenable to revascularization, native myocardial recovery has not been observed after implantation of an assist device as destination therapy. The hybrid approach of implanting autologous bone marrow stem cells during assist device implantation may eventually improve native cardiac function, which may be associated with a better prognosis eventually ameliorating the need for subsequent heart transplantation. The aforementioned hypothesis has to be tested with well-designed prospective multicentre studies.

## Introduction

Left ventricular assist devices (LVADs) are increasingly used as "bridge to transplantation" in patients with end-stage heart failure (HF) or more recently as destination therapy in non-transplant candidates. Encouraging results with LVADs as a "bridge to recovery" have been reported from the Berlin group in patients with idiopathic dilated cardiomyopathy (IDCM) [1] and by Simon and colleagues in patients with peripartum cardiomyopathy and acute myocarditis [2]. Combination therapy utilising LVADs and drug therapy, as reported by the Harefield group, has been successfully tested in non-ischemic HF patients [3]. However, myocardial recovery after mechanical support rarely occurs in the severely failing ischemic heart [2]. Ischemic cardiomyopathy (ICM) has the distinctiveness of irreversible myocardial damage with scar tissue formation and mainly impaired perfusion of the remaining viable myocardium. Myocardial remodelling process encompasses structural and molecular changes within the viable myocardium resulting from activation of mechanical, neurohormonal, and humoral reflex cascades [4]. This complex process leads to progressive changes in ventricular size, shape, and function related to cardiomyocyte hypertrophy, loss of myocytes (necrosis and apoptosis), and increased interstitial fibrosis [5].

Hibernation plays a key role in patients with coronary artery disease (CAD). Rahimtoola first described the condition of chronic sustained abnormal contraction in patients who have CAD which is reversible with revascularization and it is attributable to chronic underperfusion as myocardial hibernation [6]. Alterations in energy metabolism, energy depletion, and down-regulation of energy turnover in the hibernating myocardium trigger and maintain contractile dysfunction, continuous tissue degeneration, and cardiomyocyte loss [7]. In this setting myocardial revascularization offers the potential for enhanced prognosis.

### Chronic ischemic heart failure epidemic. Emergence of "destination therapy"

It is estimated that 6-10% of people over the age of 65 suffer from symptomatic HF in developed countries. In the USA and UK there are about 25,000 and 12,000 patients, respectively, aged less than 65 years, with severely symptomatic New York Heart Association (NYHA) class IV heart failure [8]. A meta-analysis performed by Gheorghiade and colleagues on 13 multicenter HF treatment trials, involving over 20,000 patients, revealed that CAD was the underlying aetiology in almost 70% of patients [9].

The prognosis for patients with chronic ischemic left ventricular (LV) dysfunction is poor, despite advances in pharmacological management. With only 2000 donor hearts available annually in the USA and 150 in the UK, LVADs provide an "off-the-shelf" solution for patients with end-stage ICM ineligible for transplant or for those wishing to avoid immunosuppression [10]. Use of axial-flow LVADs in large cohorts of patients deemed unsuitable for transplantation offers promising results in terms of symptomatic relief, morbidity, and mortality rates [11]. Mechanically supported hearts also demonstrate improved intrinsic myocardial contractile properties [12]. Regarding ICM, LVAD support cannot lead to repopulation of the infarcted tissue with contracting cardiomyocytes. This fact could explain the inability to wean mechanical support in patients with ICM [2].

Induction of molecular and cellular changes in the failing myocardium has been observed with the use of LVADs [13]. In an attempt to reperfuse and improve contractility to terminally ischemic myocardium we have employed a hybrid approach implanting a long-term LVAD along with injecting directly autologous bone marrow mononuclear stem cells (BMSCs) into the hibernating myocardium. Our aim is to enhance native myocardial recovery with the use of stem cells while the heart is off-loaded with the assist device.

### Our initial experience

**Figure 1:**
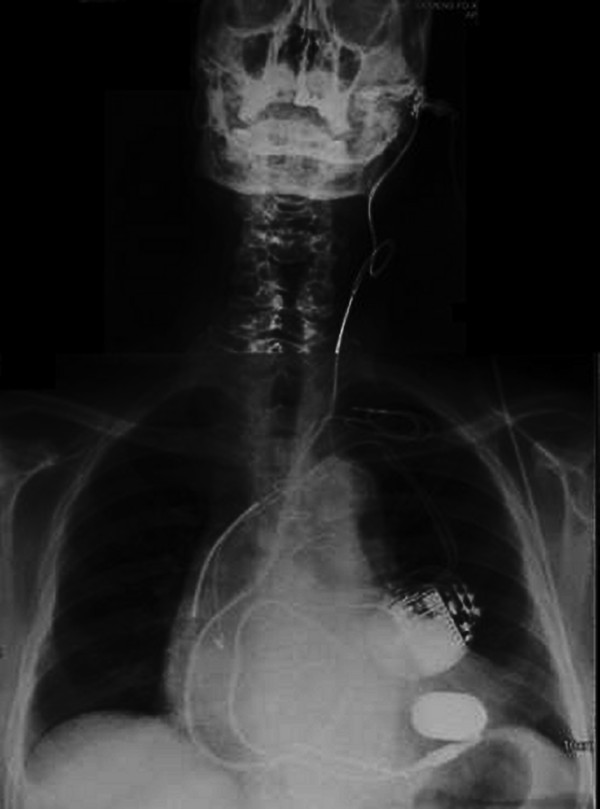
**A full range of cardiac support technology**. The plain chest x-ray shows a Jarvik pump in the apex of the left ventricle with power cable passing through the neck to the skull pedestal. There is an implantable cardio-defibrillator and dual chamber pacemaker with additional wire for cardiac resynchronisation therapy. There are drug eluting stents in the left coronary artery. Bone marrow stem cells now add a further dimension to supportive therapy.

**Figure 2:**
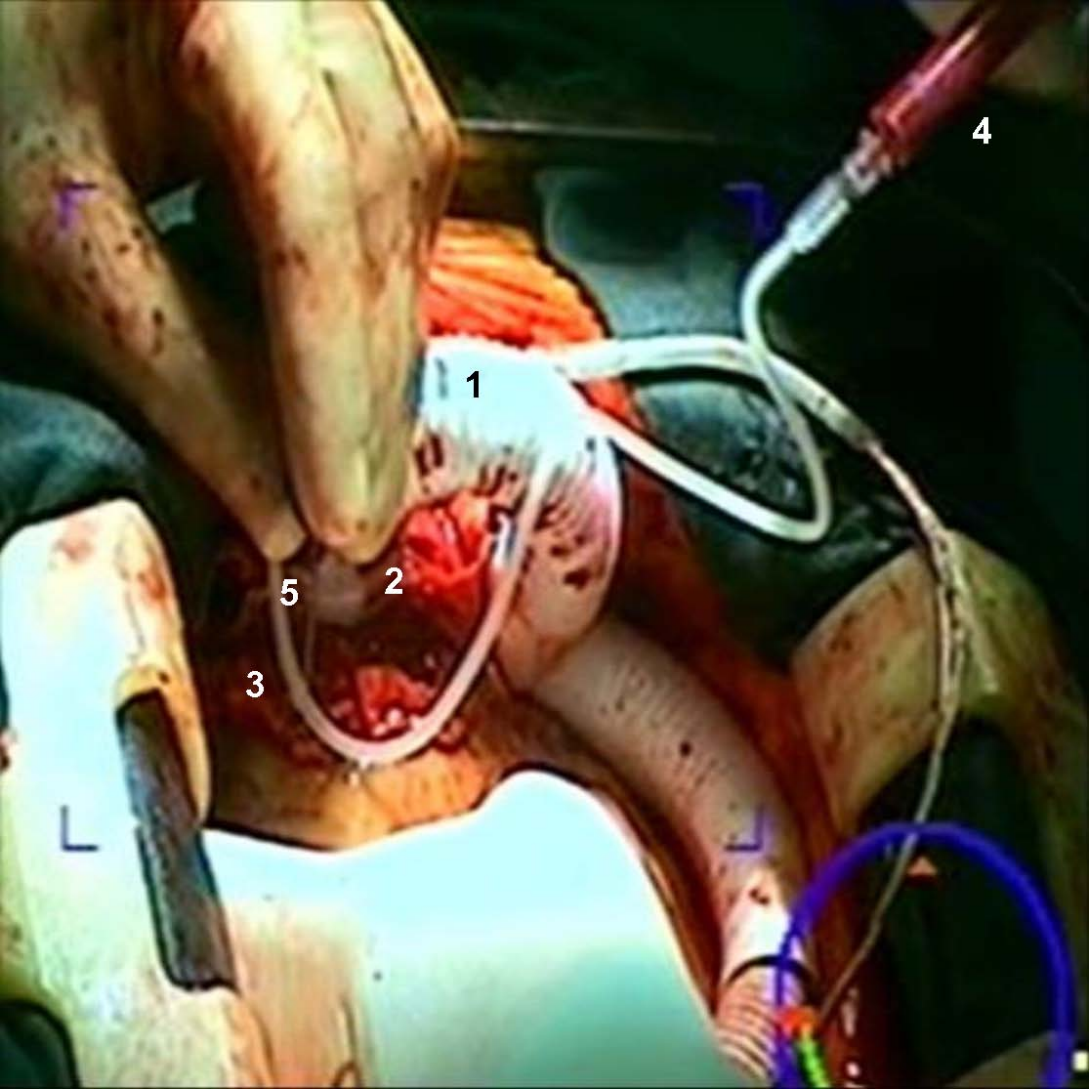
**Intraoperative view showing clinical application of stem cells into the failing heart with multiple targeted injections following device insertion**. Note the outflow graft (1) connected to the device (2) which has been implanted into the left ventricular apex (3). Stem cells injectate (4) was administered through a small needle (5) into the myocardium.

We challenged this hybrid approach in two severely symptomatic patients suffering from ICM who were hospitalized due to recurrent pulmonary oedema on minimal effort requiring intermittent inotropic support (INTERMACS level 3). They were both considered ineligible for heart transplantation due to severe co-morbidities. Autologous BMSCs were collected from bilateral anterior iliac crests during the same anaesthetic for device implantation and treated as previously described. A Jarvik 2000 axial-flow pump with skull pedestal power delivery was implanted for long-term mechanical circulatory support (Figure 1). A stem cells injectate including a mixed population of endothelial progenitor cells (CD133^+^), haematopoietic stem cells (CD34^+^), and mesenchymal stem cells (CD105^+^) was administered at pre-defined myocardial territories designated as hibernating myocardium on preoperative radionuclide scintigraphy segmental mapping (Figure 2). Recovery was uncomplicated. One patient who has completed a 12-month follow-up period is on NYHA I clinical status, while thallium scintigraphy showed functional improvement of the myocardium which could be attributed to improved reperfusion of the targeted tissue supported with autologous stem cell implantation. Current evidence on myocardial perfusion after long-term mechanical circulatory support indicates that no significant change in relative myocardial perfusion should be expected with increasing LVAD support, mainly due to cardiac autoregulatory mechanism. Therefore, transplanted stem cells provide a potential angiogenic source that could counteract this effect [14].

### Role of stem cell therapy in ischemic heart failure

Ischemic heart disease remains a major health care challenge, and progenitor cell-based therapy holds potential for treating the spectrum of myocardial ischemia. Current therapy for HF is based on the traditional belief that the heart is unable to generate new cardiomyocytes to replace failing or dying ones, but instead adapts to new stresses by myocyte hypertrophy and cardiac remodelling. Replacement of scared tissue and regeneration of viable myocardium remains a challenging target of cell transplantation therapy. However, myocardial regeneration in human has not yet been identified. Even though Orlic D, et al. reported that the injected bone marrow (BM) stem cells differentiated in a mouse myocardial infarction model into cardiomyocytes that reduced infarct size and improved myocardial function [15], Murry C, et al. showed that the injected BM stem cells very rarely, if ever, do they differentiate into cardiomyocytes [16]. Even though recent studies have challenged this conventional view by demonstrating some degree of myocardial regeneration from the native heart tissue, there is a diverse implication of regeneration among scientists [17]. Research focused on the mechanism of action of stem cells in the ischemic myocardial environment revealed that cardiac repair is promoted through paracrine activity, cell fusion, passive mechanical effects, and stimulation of endogenous repair by resident cardiac stem cells (CSC) [18].

Human heart possesses a CSC pool which is reduced in heart failure due to apoptosis, resulting in a reduced number of functionally competent cells [17]. Therefore, formation of myocytes and coronary vasculature cannot counteract the chronic loss of functional cells and vascular structures [5]. This negative balance between myocardial regeneration and loss leads to progressive ventricular dilation and deterioration of ventricular performance. Myocardial regeneration after infarction could be promoted through multifaceted cell-cell interactions between the injected stem cells and resident CSC which stimulate endogenous repair mechanisms [19].

Whilst originally intended to supply new functional cardiomyocytes, it is now clear that implanted cells respond to their environment by secreting cytokines and growth factors which act both in an autocrine fashion on the donor cells and exert paracrine effects on the host cells [18]. This process stimulates vasculogenesis and angiogenesis [20]. Moreover, transplanted BMSCs exert anti-fibrotic effects through regulation of cardiac fibroblasts proliferation and transcriptional down regulation of collagen syntheses [21]. Traditional theory that transplanted stem cells transform into new, functioning cardiomyocytes improving cardiac performance is inferential. Whether this therapy can achieve reverse remodelling and improve LVEF in the chronically ischemic heart remains unclear. The low percentage of adult stem cells in the bone marrow, low delivery efficiency, variable engraftment, and poor survival of the implanted cells in the host myocardium limit their potential for a significant clinical benefit.

### Hybrid combination therapy with LVAD and stem cells implantation; enhanced myocardial reperfusion improves prognosis

Attempts to improve cardiac performance in chronic ischemic HF patients using cell transplantation and mechanical assistance have been reported using autologous skeletal myoblasts and BMSCs [22,23]. Cell based therapy already appears to improve longevity in IDCM destination therapy patients [24]. Significant improvement in native cardiac function has been observed early after LVADs implantation attributed mainly to ventricular unloading [25]. Theoretically, LVAD unloading could reduce stem cell attrition rate by greatly reducing LV wall tension and improving myocardial perfusion [4]. Thus, whilst the blood pump provides early symptomatic improvement, stem cells may eventually provide the synergistic benefit of improving ventricular function through vasculogenesis and angiogenesis. An important finding is that over time native cardiac function deteriorated, despite histologic improvement [25]. Cell transplantation provides a promising tool in a strategy targeted at preserving improved native cardiac function during LVADs support over the long-term. This could translate in an increased potential for myocardial recovery leading to a survival benefit.

In order to test the hypothesis of myocardial reperfusion with this hybrid approach, detailed myocardial segmental viability studies as well as LV contraction analysis are essential to establish the efficacy of the method. Since the net "healing" capacity of BMSCs is difficult to determine, imaging of transplanted stem cells is crucial in order to investigate the attitude of the engrafted stem cells to the hosting myocardium [26]. The number of treated patients with the combined approach so far is limited and current evidence comes from small cohort studies or case reports that lack randomization and comparison with a control group. Another major drawback in elucidating the role of stem cell therapy in HF is that each cell-based study uses a unique protocol regarding the optimal cell type, the number of cells to be delivered, and the most suitable route for cell delivery. Design of a large multicenter randomised controlled trial with a standardized protocol is imperative in order to assess the safety and efficacy of the proposed hybrid approach in end-stage ICM.

## Conclusions

Although cellular recovery and improvement in ventricular function are evident during LVAD support in non-ischemic cardiomyopathy, the degree of cardiac recovery is limited in patients with ICM. Implantation of stem cells to promote myocardial perfusion during mechanical support in end-stage ICM may eventually provide a realistic alternative to cardiac transplantation allowing scarce donor hearts to be used for more complex cardiac defects. This hypothesis has to be tested through further well-designed randomized controlled studies.

## Competing interests

The authors declare that they have no competing interests.

## Authors' contributions

KA Conception and design, provision of patients, data analysis and interpretation, manuscript writing. PA Conception and design, data analysis and interpretation, manuscript writing. HA Data analysis and interpretation. GK Collection and assembly of data. AD Collection and assembly of data. AK Data analysis and interpretation, collection and assembly of data. CP Conception and design, data analysis and interpretation. SW Conception and design, data analysis and interpretation, manuscript writing. All authors read and approved the final manuscript.
